# Aging-Simulation Experience in Dental Education: Impact on Attitude and Empathy of Dental Students Towards Older People

**DOI:** 10.3390/dj14040224

**Published:** 2026-04-09

**Authors:** Martina Frigerio, Nattida Charadram, Mohammad Qurashi, Najla Chebib, Frauke Müller

**Affiliations:** Division of Gerodontology and Removable Prosthodontics, University Clinics of Dental Medicine, University of Geneva, 1211 Geneva, Switzerland; nattida.cha@gmail.com (N.C.); najla.chebib@unige.ch (N.C.); frauke.mueller@unige.ch (F.M.)

**Keywords:** aging-simulation experience, aging suit, undergraduate gerodontology education, teaching methodology, empathy, attitude

## Abstract

**Background:** Empathy and a positive attitude are essential competencies in healthcare, particularly when caring for older adults. Their development is therefore a critical component of undergraduate dental education. This study evaluates whether using aging simulation suits can enhance empathy and improve attitudes toward older adults among dental students. **Methods:** Third- to fifth-year dental students from the University Clinics of Dental Medicine in Geneva, Switzerland, participated in an aging-simulation experience using the GERonTologic age simulation suit (GERT^®^), which replicates age-related physical impairments. Students performed tasks in four predefined scenarios, both with and without the suit. Changes in empathy and attitudes were measured using the Jefferson Scale of Empathy-Health Profession Students’ version (JSE-HPS) and the Geriatric Attitudes Scale (GAS) questionnaires. Perceptions of the intervention were evaluated using a 5-point Likert scale questionnaire. **Results:** Sixty-three undergraduate students (45 women, 18 men), aged 20–53 years, participated. The suit significantly impaired their physical abilities (*p* < 0.001). Empathy improved post-intervention (*p* = 0.038), particularly in the third-year group. Attitude towards older adults improved significantly post-intervention (*p* = 0.001), mainly among fourth- and fifth-year students. All participants endorsed the positive value of the intervention. **Conclusions:** The intervention’s impact varied by clinical experience: empathy increased mainly in less experienced students, while attitudes improved in those with more exposure to elderly care. This suggests that the timing of simulation within the curriculum influences outcomes. Aging simulation represents a promising educational approach to enhance empathy, improve attitudes toward older adults, and prepare dental students for the clinical and psychosocial aspects of geriatric care.

## 1. Introduction

Empathy and the attitude of healthcare practitioners toward their patients are fundamental components of the practice of medicine. Empathy can be defined as the ability to sense, feel, and understand another person’s emotions [1]. It is a multidimensional concept that encompasses cognitive, emotional and behavioral components [2]. Attitude refers to the way these feelings may influence our responses in either a favorable or unfavorable manner [3]. Both have been shown to correlate with patient satisfaction, reduced anxiety and improved clinical outcomes [4,5]. Especially when treating older patients, healthcare providers need to draw on their full capacity for empathy and maintain a positive attitude toward these already vulnerable individuals. As societies continue to age, it becomes imperative to combat stereotypes and discriminatory attitudes often directed toward older adults, a phenomenon known as “ageism” [6]. This widespread bias can hinder the quality of care and support older individuals receive, affecting their physical and psychological health [7,8,9]. In this context, dental professionals are increasingly required to manage not only the clinical complexity but also the psychosocial aspects of geriatric care. However, traditional dental education has historically focused more on technical competencies, with less emphasis on broader interpersonal and reflective skills [10,11].

Research has demonstrated that empathy can be taught and strengthened through structured learning experiences [12]. For this reason, undergraduate medical and dental curricula increasingly emphasize the importance of targeted interventions to enhance empathy and attitude toward patients [13,14,15,16,17,18]. Numerous studies have explored ways to enhance empathy and attitude, employing a wide range of approaches such as role-playing, case discussions, mentoring, aging simulations, gamified aging experiences, video observations, and, more recently, technology-based methods including virtual reality. These interventions have shown varying degrees of effectiveness in fostering empathy and understanding among future healthcare professionals [19,20]. Among these interventions, aging simulation suits represent a promising tool to bridge the gap between theoretical knowledge of aging and the lived experience of older adults [21,22].

This study aimed to assess the effectiveness of aging simulation suits in replicating the physical and sensory limitations associated with aging as well as their impact on enhancing empathy and attitudes among dental students toward older adults. It also explored students’ perceptions of the relevance and value of this type of educational intervention. We hypothesized that participation in the simulation would impair performance in physical tasks and increase empathy and attitude toward older adults, while the null hypothesis was that the intervention would have no effect on these outcomes.

## 2. Materials and Methods

Third- to fifth-year undergraduate dental students from the University Clinics of Dental Medicine, University of Geneva, Switzerland, who enrolled between September 2021 to May 2022 were invited to participate in this study. The objectives and scope of the intervention project were explained during one of the undergraduate lectures and students were invited to participate. Participation took place on a voluntary basis and written consent was obtained. No formal a priori sample size calculation was performed, as this was an exploratory educational study and we aimed to include all eligible undergraduate dental students available during the study period. Given the limited number of students within the program, all volunteers were included. According to the Geneva Ethics Committee (CCER), this study did not require formal ethical approval, as it did not fall within the scope of the Swiss Human Research Act (BASEC reference number: Req-2021-00788). Additional written informed consent for publication of images was obtained from participants appearing in the figures.

### 2.1. Physical Abilities

The GERonTologic age simulation suit GERT^®^ (Product + Project, Wolfgang Moll, Andreasweg 7, 89168 Niederstotzingen, Germany) was employed for this experimental study. It consists of several separate components aiming to reproduce the impairments of sensorimotor skills associated with aging. All simulation accessories used in the different workstations, including mobility-restricting elements, noise-attenuating headphones, tremor-inducing gloves and cataract-simulating glasses, were components of the GERT^®^ age simulation suit. Four aging experiences encompassed four distinct workstations designed to replicate specific age-related challenges in this study.

#### 2.1.1. Motor Skills

**Figure 1:**
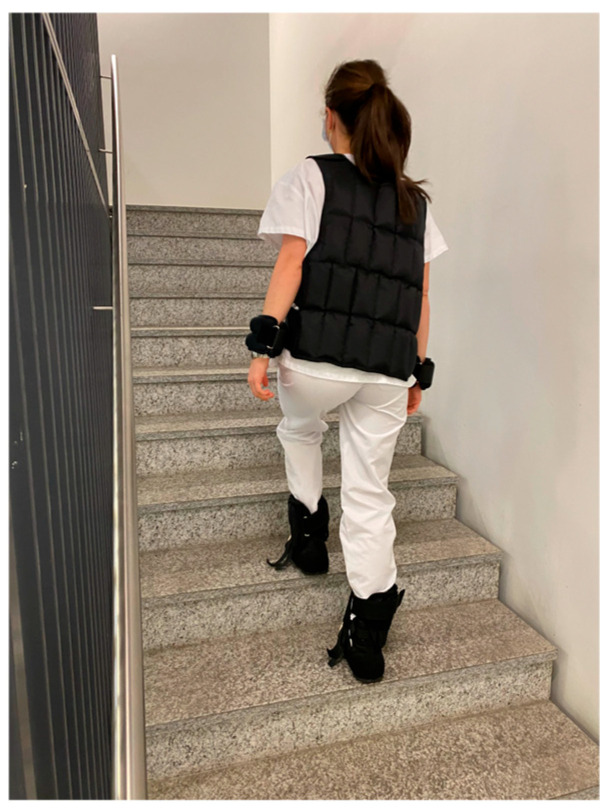
Motor skill assessment using the mobility-restricting elements of the GERT^®^ simulation suit. Participants performed a stair-climbing task to simulate reduced mobility associated with aging.

Participants were equipped with accessories to simulate reduced mobility. These accessories comprised a pair of overshoes simulating an unsteady gait, a pair of ankle guards (2 × 2.3 kg), a pair of wrist weights (2 × 1.5 kg), and a heavy jacket (10 kg). The task involved ascending and descending a total of 50 steps (Figure 1). The exercise’s timings were recorded using a stopwatch, comparing completion times with and without the aging-simulation suit.

#### 2.1.2. Hearing

**Figure 2:**
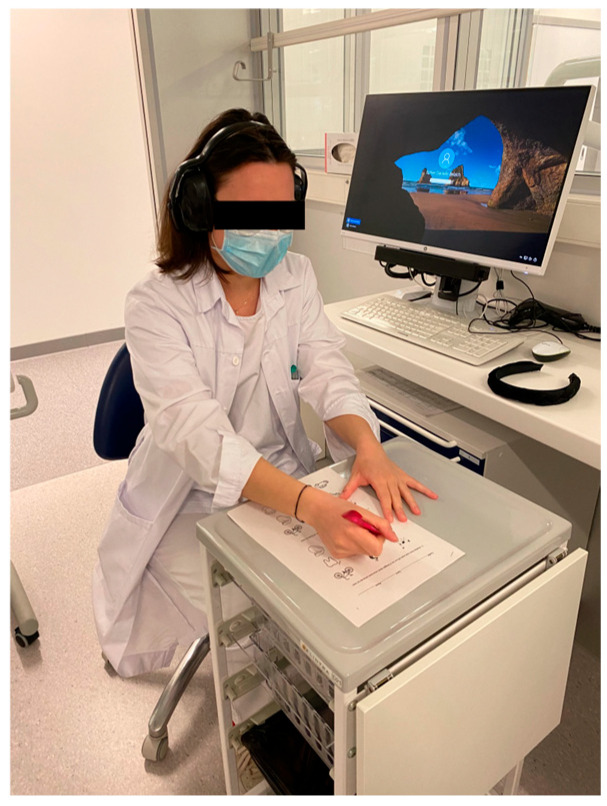
Hearing assessment while wearing anti-noise headphones (GERT^®^). Participants were asked to identify spoken words.

Participants wore anti-noise headphones and listened to a recorded voice at the same volume and distance in a quiet environment. In front of them was an A4 sheet displaying nine simple drawings of common objects (Figure 2). The recording pronounced three words, each corresponding to one of the drawings. Participants were asked to identify the words they heard by selecting the matching images. This process was repeated three times, each time with a new set of words and a different sheet of drawings. The number of correct answers was recorded.

#### 2.1.3. Tactile Sensitivity

**Figure 3:**
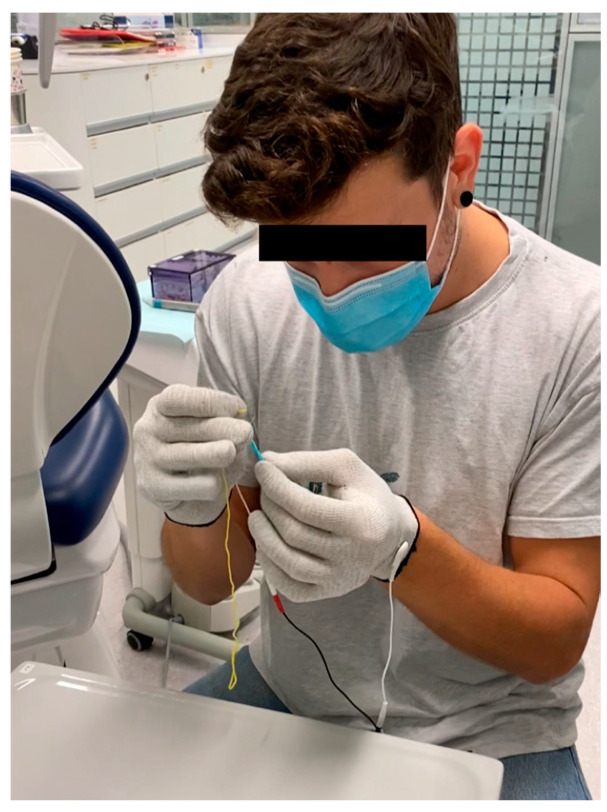
Tactile sensitivity assessment using tremor-simulating gloves (GERT^®^). Participants performed a fine motor task by threading a needle.

Students were asked to wear gloves that induced a simulated tremor. The task involved threading a sewing thread through the eye of a plastic sewing needle (Figure 3). The tremor-inducing device was set to an impulse frequency of 10 (available settings: 10, 50, or 100), and the intensity level was gradually increased to approximately 50% of the device’s maximum output and then kept constant for all participants to ensure standardized testing conditions. The time taken to complete this task with and without activating the tremor device was measured using a stopwatch. If the participant failed the test, the experiment was stopped after 1 min.

#### 2.1.4. Eyesight

**Figure 4:**
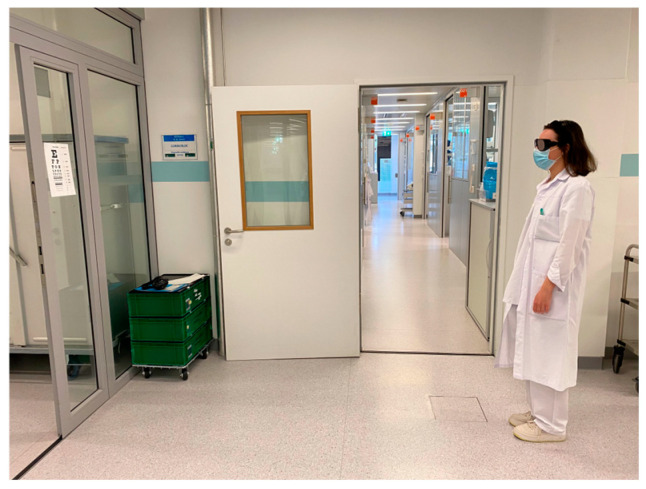
Visual assessment using cataract-simulating glasses (GERT^®^). Visual acuity was evaluated using a Snellen chart.

Special eyeglasses were used to mimic a typical visual impairment in older adults, called cataract. The visual impairment after the application of the cataract-simulated eyeglasses was evaluated using the Snellen vision test chart, a standardized visual acuity test widely employed in clinical practice. The Snellen chart used in this study was a standard A4 format, designed to be read from approximately 2.7 m (nine feet). It contains eleven lines with block letters; the first line presents the most significant letter, and the letter size gradually reduces from the second to the eleventh lines. The participants faced the Snellen chart and read aloud letters from the top to the bottom line, with and without eyeglasses (Figure 4). The last line number in which the participants successfully recognized every letter was recorded with and without eyeglasses. The score zero was given in case participants could not identify any letter on the chart.

### 2.2. Empathy and Attitude Questionnaires

Participants were asked to complete three questionnaires one week prior to the simulation experiments. The first questionnaire collected demographic information, including age, gender, presence of systemic disease, and any non-corrected visual or auditory impairments. The second questionnaire was the Jefferson Scale of Empathy-Health Profession Students’ version (JSE-HPS), used with permission from Thomas Jefferson University, which holds the copyright of the instrument [23,24]. The third was the University of California Los Angeles Geriatric Attitudes Scale (UCLA-GAS) developed by Reuben et al. [25]. Both the JSE-HPS and UCLA-GAS questionnaires were repeated after the simulation to assess changes in empathy and attitudes.

#### 2.2.1. Jefferson Scale of Empathy-Health Profession Students’ Version Questionnaire

All participants completed the 20-item JSE-HPS questionnaire one week before the experiment. Students rated one to seven on a 7-point Likert scale in response to each item (1 = strongly disagree, 7 = strongly agree). The participants were asked to complete the questionnaire one more time after the age simulation experiment. The scale was reversed for the negatively phased items. The total self-rated empathy scores obtained before and after intervention were calculated.

#### 2.2.2. University of California Los Angeles Geriatric Attitudes Scale Questionnaire

Before the intervention, students were asked to complete the UCLA-GAS questionnaire. The questionnaire contains 14 items evaluating the attitudes toward older adults and their care. Participants rated items one to five (1 = strongly disagree, 5 = strongly agree) on a 5-point Likert scale. After completing the age simulation experiment, participants were asked to complete the questionnaire again. Scores on the negatively worded statements were reversed before they were added to scores on the positively worded statements to produce a total score.

### 2.3. Usefulness and Feasibility of Aging Simulation Intervention

The appropriateness of this type of educational intervention for the undergraduate curriculum was evaluated using a 5-point Likert scale (1 = strongly disagree, 5 = strongly agree) questionnaire. Five questions covered the usefulness of the geriatric education tool and assessed if the simulation was perceived as “realistic”, if it provided an “unthreatening” learning environment, if it would improve attitude and empathy towards older patients, and if it should be integrated into the undergraduate curriculum.

### 2.4. Protocol

The experiment took place at the University Clinics of Dental Medicine of Geneva, Switzerland, outside of clinical hours. The simulation sessions were conducted on three proposed dates between April and May 2022. Upon arrival, participants handed in the pre-intervention forms they had received and completed the week prior to the simulation.

All sessions were conducted at the same time of day, immediately after clinical activities, to minimize variations in fatigue or alertness. Each station lasted approximately 4–5 min, and participants completed all four stations within a total duration of approximately 20 min, after which participants were given additional time to fill in the post-intervention questionnaires.

Each simulation station was supervised by a designated facilitator who assisted students in putting on the simulation equipment and provided standardized instructions. Participants were given the same explanations prior to each task to ensure consistency of the experimental conditions. The sequence of stations was randomized to avoid order effects.

The experimental setup and procedures were designed to be easily reproducible in similar educational settings.

### 2.5. Statistical Analysis

Data obtained from the experiment were analyzed using SPSS Statistics version 27 (IBM Corp., Armonk, NY, USA). Mean and standard deviations from questionnaires before and after the experiments were recorded and compared. The Shapiro–Wilk test evaluated the normality of the data. The paired *t*-test was used to compare the data before and after the aging suit experiment, and between the different subgroups of participants (third year, fourth year, and fifth year dental students). A Wilcoxon signed ranks test was used where the data deviated from a normal distribution. A *p* < 0.05 was considered to indicate statistical significance.

## 3. Results

A total of 63 undergraduate dental students participated in the study, comprising 23 third-year students, 17 fourth-year students, and 23 fifth-year students. The mean age of participants was 25.0 years (SD = 6.2) and ranged from 20 to 53 years. The sample included 45 women (71.4%) and 18 men (28.6%). All participants were in good general health, with no reported systemic diseases or physical impairments.

### 3.1. The Alteration of Physical Abilities

#### 3.1.1. Motricity Test

**Figure 5:**
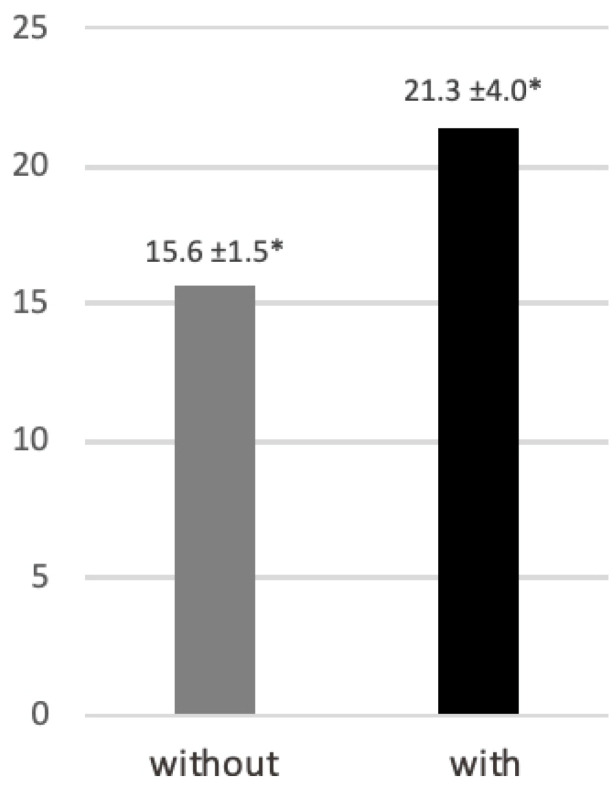
Time (seconds) required to ascend and descend stairs without and with the aging-simulation suit. Data are presented as mean ± standard deviation. * *p* < 0.05.

When applying the aging simulation suit, participants required significantly more time climbing up the stairs than without the suit (21.3 ± 4.0 s vs. 15.6 ± 1.5 s; *p* < 0.05) (Figure 5).

#### 3.1.2. Auditory Test

**Figure 6:**
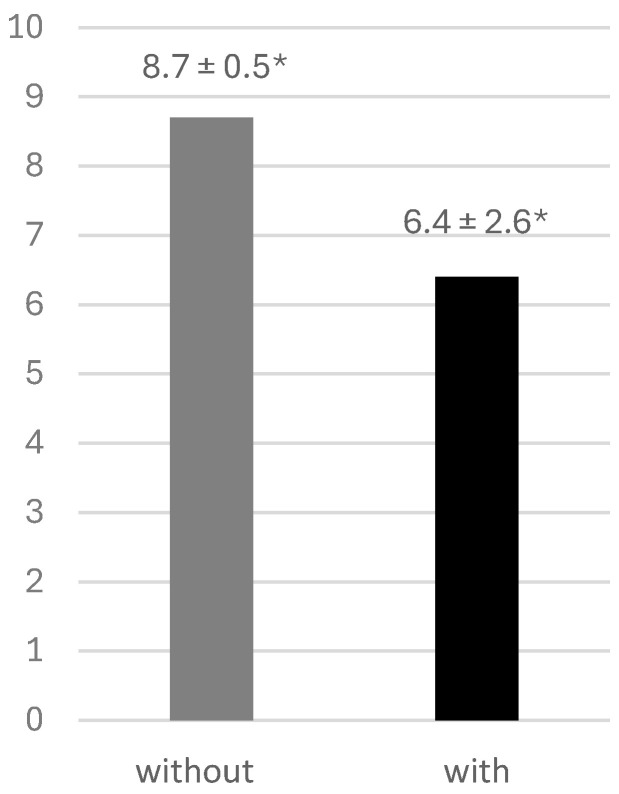
Number of correctly recognized spoken words without and with the anti-noise headphones. Data are presented as mean ± standard deviation. * *p* < 0.05.

The anti-noise headphones significantly reduced the ability to hear the registered spoken words (6.4 ± 2.6 words) in contrast to when not wearing the headphones (8.7 ± 0.5 words), confirmed by *p* < 0.05 (Figure 6).

#### 3.1.3. Tactile Sensory Test

**Figure 7:**
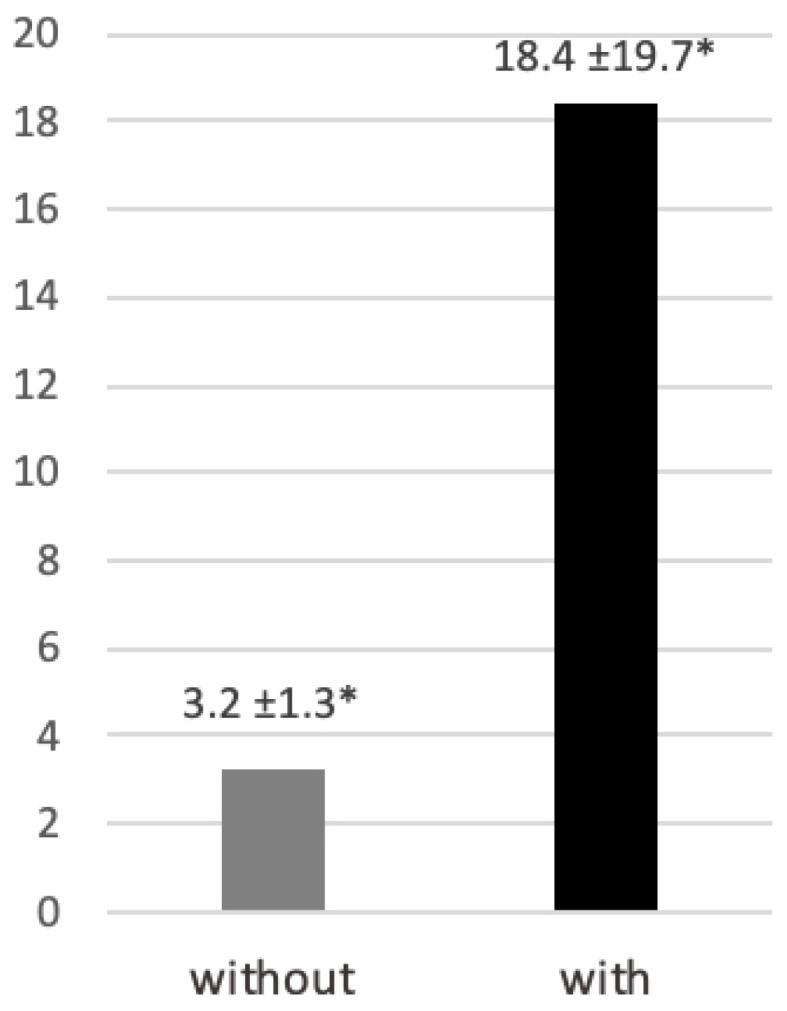
Time (seconds) required to thread a needle without and with tremor-simulating gloves. Data are presented as mean ± standard deviation. * *p* < 0.05.

Participants experienced difficulty controlling their hands while threading a needle when wearing gloves to simulate tremors. Time spent on the task increased significantly compared with the condition without gloves (18.4 ± 19.7 s vs. 3.2 ± 1.3 s; *p* < 0.05) (Figure 7).

#### 3.1.4. Vision Test

**Figure 8:**
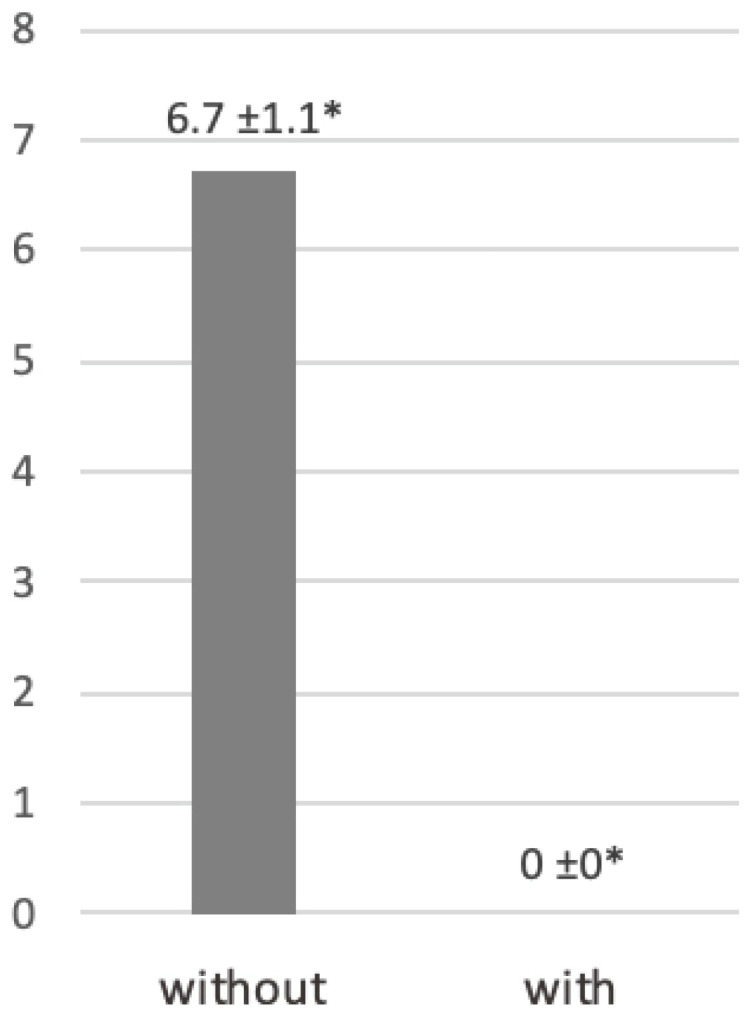
Visual acuity performance (line number) assessed with the Snellen chart without and with cataract-simulating eyeglasses. Data are presented as mean ± standard deviation. * *p* < 0.05.

The eyeglasses imitating cataracts impeded the participants’ vision, as they could not read any letter on the Snellen chart. Overall, the mean score without eyeglasses was 6.7 ± 1.1. The difference was statistically significant (*p* < 0.05) (Figure 8).

### 3.2. Empathy and Attitude Towards Older People

#### 3.2.1. Empathy

The JSE-HPS score increased significantly among all students after the intervention (*p* = 0.038). However, when analyzing empathy by subgroup (third-, fourth-, and fifth-year dental students), only the third-year students showed a statistically significant improvement (*p* = 0.026). Detailed results are presented in Table 1.

#### 3.2.2. Attitude

According to the score obtained from the UCLA-GAS, the overall attitude towards older people improved significantly after the experiment (*p* = 0.001). The analysis revealed that this improvement was significant in the fourth- (*p* = 0.043) and fifth-year (*p* = 0.043) dental students (Table 2).

### 3.3. Appropriateness of the Intervention for the Dental Curriculum

Participants unanimously agreed that including this intervention in the pre-graduate curriculum in dentistry would be helpful and improve the empathy and attitude towards older persons. Evaluation of the 5-point Likert scale questionnaire regarding the usefulness and feasibility of this aging simulation experiment after the simulation is presented in Table 3.

## 4. Discussion

This study assessed the impact of an aging simulation suit on empathy and attitudes toward older adults among undergraduate dental students at different stages of their curriculum. We measured both the physical effectiveness of the suit in simulating age-related limitations and its influence on students’ perceptions.

The results of the intervention confirm the effectiveness of the aging simulation suit GERT^®^ in realistically reproducing age-related physical limitations in a healthy young population. The suit successfully simulated key sensory and functional declines, including reduced motor coordination, visual and auditory acuity, and tactile sensitivity, which older adults commonly experience. This validation of the simulation suit provides a solid foundation for its use in educational settings aimed at enhancing healthcare students’ understanding of aging-related challenges. Previous studies have also confirmed the validity of aging simulation suits in reproducing the physical effects of aging [16,21,22,26]. To ensure a reliable assessment of the suit’s effectiveness, the scenarios implemented in this study were deliberately standardized and controlled.

Participation in the aging simulation activities led to improved empathy and attitudes toward older adults among dental students. Our findings are consistent with previous studies showing that simulation-based interventions can positively influence these dimensions [16,19,27,28,29]. However, not all studies have reported positive outcomes; in some cases, simulation experiences have been associated with increased anxiety or discomfort, potentially leading to more negative attitudes or less empathy [14,30]. In our study, empathy significantly improved among third-year dental students following the intervention. This change was not observed among fourth- and fifth-year students, whose empathy scores were already higher at baseline and remained stable after the simulation. This discrepancy likely reflects the differing levels of clinical experience across groups. While third-year students typically have no direct contact with older patients, fourth- and fifth-year students have already been exposed to geriatric care during their clinical rotations. Repeated interaction with older adults in real clinical contexts may contribute to the development of empathic attitudes, which the aging simulation suit then reinforces rather than initiates. In contrast, for third-year students, the simulation may have provided a novel and impactful first opportunity to engage with the physical and emotional challenges of aging, thereby triggering a more pronounced shift in empathy.

Regarding attitudes, the evolution followed an opposite pattern. A statistically significant improvement in attitudes toward older adults was observed only among fourth- and fifth-year students. One possible explanation is that, although these students already possess some level of empathic understanding through clinical exposure, the simulation enabled them to embody the aging process in a more personal and tangible way. Being placed in the physical situation of older adults may have deepened their appreciation of the lived experience of aging, reinforcing respectful and compassionate attitudes. Conversely, for third-year students who had not yet engaged in the clinical care of older adults, the simulation may have elicited anxiety or discomfort when confronted with unfamiliar physical decline, which could interfere with the formation of a more positive attitude. This suggests that the stage at which the simulation is introduced, whether before or after substantial patient contact, can shape how students perceive and internalize the experience.

All participating students reported that they valued the experience and supported the integration of aging simulation into the dental curriculum. They recognized its potential to enhance awareness, sensitivity, and preparation for geriatric care. Undergraduate dental education traditionally places strong emphasis on the technical and biomedical aspects of treating older adults, including management of medical complexity, polypharmacy, prosthodontic rehabilitation, and adaptations of clinical procedures to age-related physiological changes [31,32,33,34]. While these competencies are essential, less curricular attention has historically been devoted to the development of empathy, attitudes, and reflective understanding of the lived experience of aging. As a result, students may become technically competent in managing older patients without being equally prepared for the psychosocial, emotional, and functional challenges that accompany aging. This imbalance becomes increasingly relevant as dental students, and future professionals worldwide, face a growing proportion of older adults in clinical settings.

This study’s strength lies in the inclusion of students across multiple academic years, which enabled comparisons between students with and those without previous clinical exposure to older patients. All simulations were conducted at the same time of day, minimizing variability in fatigue or alertness across participants. However, several limitations must be acknowledged. The study was conducted in a single institution with a relatively small sample size, which may limit generalizability. Educational systems, sociocultural contexts, and attitudes towards aging may vary across universities, countries and healthcare settings. These differences should be taken into account when interpreting and generalizing the present findings. Furthermore, the age range among participants was relatively broad (20–53 years), although the majority of students were in their early twenties. The higher variability observed in the fourth-year group was due to the presence of a small number of older participants. Given their limited number, no meaningful subgroup analysis based on age could be performed. In addition, previous research in a Swiss multicenter study found no significant effect of demographic variables on attitudes toward older adults [35]. Another limitation is that the intervention consisted of a single simulation session and did not assess long-term effects. It therefore remains unclear whether the observed changes in empathy and attitude persist over time and translate into long-term, behavioral changes. The positive responses may partly reflect an immediate positive reaction to a novel and immersive learning experience. Longitudinal studies are needed to assess the durability of these effects. Furthermore, empathy and attitude were evaluated using self-reported questionnaires. Although validated instruments were used, such measures may be influenced by social desirability bias, meaning that students may tend to respond in a manner they perceive as expected or socially acceptable.

Although the intervention was associated with improvements in self-reported empathy and attitudes, the present study did not assess whether these changes translate into observable behavioral changes in clinical practice. Future research should investigate the impact of such interventions on patient interaction, communication, and quality of care.

The findings of the present study suggest that experiential learning approaches, such as aging simulation, can complement technical training with embodied understanding, thereby fostering more compassionate and patient-centered care. Integrating such interventions alongside conventional clinical teaching may be particularly important to counteract ageism and to prepare future dentists for the complex realities of geriatric dental care. Careful integration into the curriculum, considering timing, complementary activities, and opportunities for structured reflection, may enhance their impact and promote more compassionate, age-inclusive care practices. While simulation-based learning shows promise, its long-term impact on clinical behavior remains uncertain. Embedding these experiences within a longitudinal curriculum and evaluating students over time may help clarify whether early attitudinal changes translate into lasting improvements in geriatric dental care.

## 5. Conclusions

By enabling undergraduate dental students to experience age-related physical and sensory limitations firsthand, aging simulation appears to be an effective approach for enhancing empathy and improving attitudes toward older adults. The intervention was well received and perceived as both feasible and educationally valuable. These findings support the inclusion of aging simulation activities in undergraduate dental curricula as a means to complement technical training and address age-related bias. However, further research is needed to determine whether such interventions lead to sustained behavioral change and improved care in real clinical settings.

## Figures and Tables

**Table 1:** Changes in students’ empathy toward older adults: JSE-HPS scores of all students, third-, fourth-, and fifth-year dental students, before and after application of aging simulation suits.

	All Students	3rd Year Students	4th Year Students	5th Year Students
**Before (mean ± SD)**	115.0 ± 9.99	113.8 ± 10.46	114.4 ± 10.49	116.6 ± 9.35
**After (mean ± SD)**	116.9 ± 10.33 *	115.9 ± 11.17 *	116.5 ± 11.30	118.3 ± 8.94

* *p* < 0.05 (Wilcoxon test).

**Table 2:** Changes in students’ attitude toward older adults: UCLA-GAS scores of all students, third-, fourth-, and fifth-year dental students, before and after application of aging simulation suits.

	All Students	3rd Year Students	4th Year Students	5th Year Students
**Before (mean ± SD)**	3.6 ± 0.45	3.6 ± 0.39	3.6 ± 0.51	3.5 ± 0.47
**After (mean ± SD)**	3.7 ± 0.46 *	3.6 ± 0.45	3.8 ± 0.45 *	3.7 ± 0.48 *

* *p* < 0.05 (paired *t*-test).

**Table 3:** Students’ level of agreement with statements related to the usefulness and feasibility of the aging simulation intervention. Values are expressed as mean ± standard deviation (SD), median, and interquartile range (IQR). Agreement was rated using a 5-point Likert scale (1 = strongly disagree, 5 = strongly agree).

QUESTIONS	MEAN ± SD	MEDIAN	IQR
Q1. Aging simulation is useful as a geriatric education tool	4.6 ± 0.8	5	1
Q2. The aging simulation scenarios were realistic	4.3 ± 0.7	4	1
Q3. Aging simulation suits provided an unthreatening learning environment	4.5 ± 0.8	5	1
Q4. Aging simulation will improve my attitude and empathy toward older patients	3.9 ± 1.0	4	2
Q5. Aging simulation should be integrated into the pre-graduation course	4.1 ± 1.0	4	1

## Data Availability

The data presented in this study are available on request from the corresponding author (the data are not publicly available due to ethical and privacy restrictions related to the participation of human subjects).

## References

[1] Hojat M., Gonnella J.S., Nasca T.J., Mangione S., Vergare M., Magee M. (2002). Physician empathy: Definition, components, measurement, and relationship to gender and specialty. Am. J. Psychiatry.

[2] Byrne M., Daly S., McCann C.M., Miles A. (2026). Components of empathy: A Delphi study. Patient Educ. Couns..

[3] Tahani B., Manesh S.S. (2021). Knowledge, attitude and practice of dentists toward providing care to the geriatric patients. BMC Geriatr..

[4] Derksen F., Bensing J., Lagro-Janssen A. (2013). Effectiveness of empathy in general practice: A systematic review. Br. J. Gen. Pract..

[5] Licciardone J.C., Tran Y., Ngo K., Toledo D., Peddireddy N., Aryal S. (2024). Physician Empathy and Chronic Pain Outcomes. JAMA Netw. Open.

[6] Ng R., Allore H.G., Trentalange M., Monin J.K., Levy B.R. (2015). Increasing negativity of age stereotypes across 200 years: Evidence from a database of 400 million words. PLoS ONE.

[7] Bowling A. (1999). Ageism in cardiology. BMJ.

[8] Neal D., Morgan J.L., Kenny R., Ormerod T., Reed M.W. (2022). Is there evidence of age bias in breast cancer health care professionals’ treatment of older patients?. Eur. J. Surg. Oncol..

[9] Hartgerink J.M., Cramm J.M., Bakker T.J., Mackenbach J.P., Nieboer A.P. (2015). The importance of older patients’ experiences with care delivery for their quality of life after hospitalization. BMC Health Serv. Res..

[10] Kossioni A.E. (2025). In need of a prosthodontics education with a focus on Healthy Ageing. J. Prosthodont. Res..

[11] Hillebrecht A.L., Barbe A.G., Krämer S., Auerbacher M., Semper-Hogg W., Spies B.C., Linde P., Erfurt-Berge C., Kossioni A., Farin-Glattacker E. (2026). Competences for Providing Oral Health Care to Care-Dependent Older Adults-Defining Learning Objectives for the German Undergraduate Dental Curriculum Through a Delphi Study. Eur. J. Dent. Educ..

[12] Riess H. (2022). Empathy can be taught and learned with evidence-based education. Emerg. Med. J..

[13] Ross L., Jennings P., Williams B. (2018). Improving health care student attitudes toward older adults through educational interventions: A systematic review. Gerontol. Geriatr. Educ..

[14] Lucchetti A.L., Lucchetti G., de Oliveira I.N., Moreira-Almeida A., da Silva Ezequiel O. (2017). Experiencing aging or demystifying myths?—Impact of different “geriatrics and gerontology” teaching strategies in first year medical students. BMC Med. Educ..

[15] Patel S., Pelletier-Bui A., Smith S., Roberts M.B., Kilgannon H., Trzeciak S., Roberts B.W. (2019). Curricula for empathy and compassion training in medical education: A systematic review. PLoS ONE.

[16] Rodriguez-Molinero J., Delgado-Somolinos E., Miguelañez-Medrán B.C., Ramirez-Puerta R., Corral-Liria I., Jiménez-Fernández R., Losa-Iglesias M.E., López-Sánchez A.F. (2024). Use of an age-simulation suit as an empathy-building method for dental students: A pre-post study. PeerJ.

[17] Pira G.L., Ruini C., Vescovelli F., Baños R., Ventura S. (2025). Could Empathy Be Taught? The Role of Advanced Technologies to Foster Empathy in Medical Students and Healthcare Professionals: A Systematic Review. J. Med. Syst..

[18] Liu J.Y.W., Mak P.Y., Chan K., Cheung D.S.K., Cheung K., Fong K.N.K., Kor P.P.K., Lai T.K.H., Maximo T. (2024). The Effects of Immersive Virtual Reality-Assisted Experiential Learning on Enhancing Empathy in Undergraduate Health Care Students Toward Older Adults with Cognitive Impairment: Multiple-Methods Study. JMIR Med. Educ..

[19] Kelm Z., Womer J., Walter J.K., Feudtner C. (2014). Interventions to cultivate physician empathy: A systematic review. BMC Med. Educ..

[20] Samarasekera D.D., Lee S.S., Yeo J.H.T., Yeo S.P., Ponnamperuma G. (2023). Empathy in health professions education: What works, gaps and areas for improvement. Med. Educ..

[21] Lavallière M., D’Ambrosio L., Gennis A., Burstein A., Godfrey K.M., Waerstad H., Puleo R.M., Lauenroth A., Coughlin J.F. (2017). Walking a mile in another’s shoes: The impact of wearing an Age Suit. Gerontol. Geriatr. Educ..

[22] Vieweg J., Schaefer S. (2020). How an Age Simulation Suit affects Motor and Cognitive Performance and Self-perception in Younger Adults. Exp. Aging Res..

[23] Hojat M., Mangione S., Nasca T.J., Cohen H.J., Gonnella J.S., Erdmann J.B. (2001). The Jefferson Scale of Physician Empathy: Development and Preliminary Psychometric Data. Educ. Psychol. Meas..

[24] Fields S.K., Mahan P., Tillman P., Harris J., Maxwell K., Hojat M. (2011). Measuring empathy in healthcare profession students using the Jefferson Scale of Physician Empathy: Health provider—Student version. J. Interprofessional Care.

[25] Reuben D.B., Lee M., Davis J.W., Eslami M.S., Osterweil D.G., Melchiore S., Weintraub N.T. (1998). Development and validation of a geriatrics attitudes scale for primary care residents. J. Am. Geriatr. Soc..

[26] Engelken M., Merritt J., Rutherford A., Smith J. (2025). The effectiveness of a geriatric simulation suit for improving empathy and simulating the aging process for older adults in a DPT program: A pilot study. Gerontol. Geriatr. Educ..

[27] Eost-Telling C., Kingston P., Taylor L., Emmerson L. (2021). Ageing simulation in health and social care education: A mixed methods systematic review. J. Adv. Nurs..

[28] Varkey P., Chutka D.S., Lesnick T.G. (2006). The Aging Game: Improving medical students’ attitudes toward caring for the elderly. J. Am. Med. Dir. Assoc..

[29] Merino López C., Díaz Rodríguez J., Bango Sánchez S., García Castaño L., García Mata P., Arribas Gonzalo E., Cernuda Martínez J.A. (2025). Clinical Simulation of Aging in Nurses and Its Impact on Their Empathy: A Cluster-Randomized Controlled Trial. Nurs. Health Sci..

[30] Bearman M., Palermo C., Allen L.M., Williams B. (2015). Learning Empathy Through Simulation: A Systematic Literature Review. Simul. Healthc..

[31] Xavier I., Ettinger R.L., Proença L., Botelho J., Machado V., Rua J., Delgado A.S., Mendes J.J. (2020). Geriatric Dentistry Curriculum in Six Continents. Int. J. Environ. Res. Public Health.

[32] Kossioni A., McKenna G., Müller F., Schimmel M., Vanobbergen J. (2017). Higher education in Gerodontology in European Universities. BMC Oral Health.

[33] Kossioni A., Vanobbergen J., Newton J., Müller F., Heath R. (2009). European College of Gerodontology: Undergraduate curriculum guidelines in gerodontology. Gerodontology.

[34] Kitagawa N., Sato Y., Komabayashi T. (2011). Graduate and undergraduate geriatric dentistry education in a selected dental school in Japan. Eur. J. Dent. Educ..

[35] Carellis C., Kalberer N., Abou-Ayash S., Schimmel M., Wittneben J.G., Zitzmann N.U., Besimo C.E., Bornstein M.M., Müller F., Srinivasan M. (2021). Attitudes of dental students towards treating elderly patients. Swiss Dent. J..

